# Environmental Sustainability of Lighter Fluids†

**DOI:** 10.1021/acsomega.3c05242

**Published:** 2024-01-17

**Authors:** Edit Cséfalvay, Viktória Kovács

**Affiliations:** Department of Energy Engineering, Faculty of Mechanical Engineering, Budapest University of Technology and Economics, Muegyetem rkp. 3., H–1111 Budapest, Hungary

## Abstract

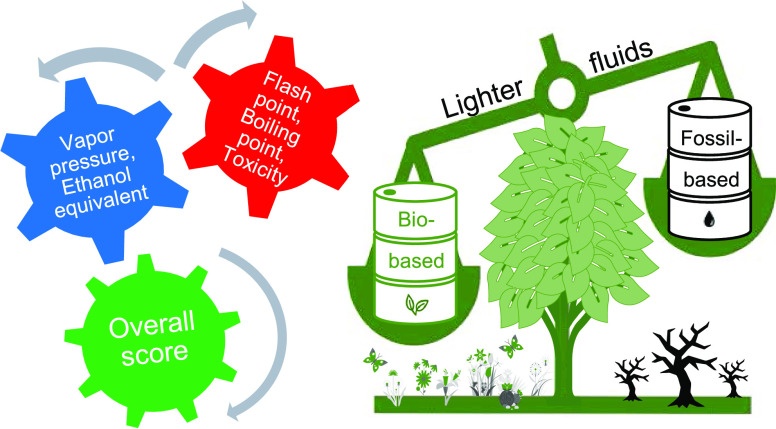

Lighter fluids are consumer products used only at a low-volume scale, representing a realizable goal of fossil fuel replacement by renewables. Physicochemical properties of four fossil-based conventional lighter fluids (Ronsonol, Zippo, Landmann, and Terracotta) and six selected biomass-based chemicals (γ-valerolactone, ethyl-levulinate, ethanol, n-butanol, γ-valerolactone 90% v/v and ethanol 10% v/v, and ethyl-levulinate 90% v/v and ethanol 10% v/v mixtures) as potential biomass-based lighter fluids were assessed. Assessments were carried out in terms of safety, toxicological, and environmental viewpoints, represented by a flash point, boiling point, vapor pressure values, and evaporation rates; oral toxicity measured on rats; and real ethanol equivalent values, respectively. Parameters were collected where available; in the absence of literature data, they were calculated or measured and then analyzed. Finally, multicriteria analysis based on the flash point, boiling point, vapor pressure, toxicity, and ethanol equivalent values revealed γ-valerolactone as a renewable substance, which can be a promising alternative to replace fossil-based lighter fluids because it was awarded as the first in the multicriteria evaluation by obtaining the highest value of the overall scores. In practical usage, however, ignition, combustion experiments, flue gas, and emission analysis are also required to underline its commercial use in the future.

## 1 Introduction

The security of supply in the energy sector and the uncertainty of the prices of fossil-based energy resources have initiated researchers and scientists to find alternative resources to maintain the continuous supply of energy generation. On the other hand, the unknown date of depletion of fossil resources and the extremely increased prices have urged researchers, scientists, and engineers to find alternative resources/raw materials that could replace the currently large-volume-used crude oil and natural gas, which represent a significant part of current carbon-based consumer products.^1^ Ethanol equivalent—introduced as a conversational tool between carbon-based chemicals and energy—calculations pointed out that fossil fuel used as a primary energy source could not be replaced entirely by renewables;^2,3^ however, the raw material replacement of fossil fuels in the chemical industry seems to be a more realistic goal when using bioethanol.^4,5^ The transition of chemical industry feedstocks from fossil-based raw materials to renewable resources has to be accelerated to establish greener, cleaner, or even sustainable production. The intensive research activity on the utilization of biobased chemicals has started for ca. five decades, and a group of possible surrogates, which could be obtained via appropriate chemical conversion of biomass, was proposed.^4^ Due to the successful research work, the production of several chemicals has been realized on a biomass basis at an industrial scale; therefore, economical production of the so-called initial platform chemicals (IPCs) has started on a biomass basis. A review was published on the conversion of biomass to IPCs, i.e., ethanol, 3-hydroxypropionic acid, propionic acid, succinic acid, furfural, levulinic acid, isoprene, and 5-hydroxymethylfurfural, focusing on industrial-scale realizations.^6^ It was concluded that the volume of consumed chemicals had to be considered as a crucial factor for this purpose. There were only a few replacements that were industrially viable fossil-based chemicals.^7^

Lighter fluids (LFs) are consumed only at the kiloton scale (Table 1); therefore, a real objective of this effort could be the replacement of hydrocarbon-based LFs with chemicals derived from biomass conversion.

**Table 1:** Consumption of Grill Lighter Fluids Labeled R65 or H304 in Different Countries

country	year	annual lighter fluid consumption	refs
Cyprus	2014	approx. 346 t	(8)
Estonia	2014	approx. 137 t	(8)
Finland	2008–2013	average 460 t	(8)
Germany	2007	approx. 3500 t	(8)
Italy	2014	approx. 400 t	(8)
Norway	2008–2013	average 1230 t	(8)
Poland	2014	approx. 1750 t	(8)
Sweden	2006	approx. 4000 t	(8)
USA	1989	46,250 t	(9)

LFs are liquid substances or mixtures that are readily ignitable by flame and can be used to ignite solid barbecue fuel, such as charcoal. When using LFs, safety must be a priority. Safe use of LFs can be supported when the LF itself is well-selected and its physicochemical properties related to safety (such as the flash point (FP), boiling point (BP), vapor pressure (VP) values, and evaporation rates (ER)) are on the “safe” side. If the LF has a high VP, by opening the LF flask, its smell can be immediately recognized and can be inhaled, which is indeed not safe. According to the safety data sheets, most of the LFs are classified into Risk Phrase R65, i.e., “may cause lung damage, if swallowed” and “may be fatal if swallowed and enters airways” (Hazard Statement H304). Therefore, the European Chemicals Agency (ECHA) published a report on fluids labeled R65 or H304, considering the trends in consumption and poisonings caused by grill LFs.

Typically, hydrocarbon-based LFs are composed of C_9_–C_14_ aliphatic hydrocarbons, mainly alkanes and alkenes, but some of them can contain aromatic constituents of up to 2% of the total volume. Traces of volatile C_5_ and C_6_ compounds are also usually utilized as additives because they facilitate fast ignition. Some LFs also contain up to 10% of short-chain alcohols, such as 2-propanol or 2-butanol, to replace volatile C_5_ and C_6_ fractions.^8^ ECHA has suggested some alternatives that are available on the market, such as denatured alcohols (such as bioethanol, propyl alcohol, or butyl alcohol) or vegetable oils (methyl esters of long-chain fatty acids). Paraffin mixtures are also viable alternative LFs. ECHA names a mixture containing 38.2% water, 60% isopropyl alcohol, 1.2% triethanolamine, and a 0.6% polymer, a product in the Greek market, but it does not represent a good lightning performance. Turning the focus on exclusively biomass-based alternatives, one can find patented LFs composed of terpene and/or terpenoid oil mixed with short-chain alcohol to initiate fast ignition,^10,11^ γ-valerolactone (GVL) 50–100 wt % and ethanol (EtOH) 0–50 wt % mixtures,^12^ and mixtures of 70–50 wt % *n*-butanol (*n*-BuOH) and 30–50 wt % biodiesel originating from transesterification of vegetable oil or animal fat mixtures.^13^ Our previous study pointed out that GVL mixed with EtOH 10% v/v and ethyl-levulinate (EL) mixed with EtOH 10% v/v can also be used as alternatives to traditional lighter fluids representing a relatively low emission.^14^

In addition to the composition of LFs, their physicochemical properties are essential. Of these, FP, BP, and VP are the most important parameters for combustion and safe storage. The perfect barbecue temperature can differ between countries, regions, and climates, but most people prefer summer for grilling. Focusing on the United States, a survey called “Outdoor & Barbecues in the United States 2017” revealed that July was the ideal month for barbecuing. June was also preferred, but August, September, and even May were also selected as a time for barbecuing.^15^ July represents the hottest month of the year: the average daily temperature—the average temperature of the hourly measured values, including nights—was 24 °C in 2017.^16^ Considering the daytime temperatures, the mean daytime temperature in the USA in July between 1901 and 2000 was 30.38 °C.^17^ It is worth noting that a special weather report for barbecue is available on the Internet where barbecue fans can check the actual or forthcoming weather.^18^ As U.K. people enjoy outdoor activities, they agree on sunny and low humidity weather and 22–25 °C temperature as the perfect weather for grilling.^19^ Considering the high daytime temperatures in July in the USA, evaporation of LFs during grilling is a key issue.

From a safety point of view, low VP favors the safe storage and low emission caused by evaporation during the preparation phase of grilling; on the other hand, it makes the ignition difficult. The physicochemical properties of selected commercially available lighter fluids such as Ronsonol and Zippo (mainly available in the US market) and Landmann and Terracotta (available in the EU market) are summarized in Table 2. Ronsonol’s VP is lower compared to Zippo’s, but no more analysis can be derived for the Landmann and Terracotta in the absence of literature data. Another requirement for LFs is that they shall be ignited easily, which is represented by a low FP, but this also exhibits danger during application. Application and storage have conflicting effects on VP and FP, and their assessment is essential. As shown in Table 2, approximate values or ranges are found in most cases in material safety data sheet (MSDS), which require clarification. BP ranges are provided for hydrocarbon mixtures as well as density ranges. A lower heating value (LHV) is not part of MSDS; however, it can provide information on combustion properties. LHVs of Ronsonol and Zippo as hydrocarbon mixtures were estimated in our previous study,^14^ but they are still needed to be determined for Landmann and Terracota LFs.

**Table 2:** Physicochemical Properties of Selected Commercially Available Lighter Fluids Labeled with R65 or H304

lighter fluid	Ronsonol^20^	Zippo^21^	Landmann^22^	Terracotta^23^
composition	blend of aliphatic hydrocarbons	mixture of 70 wt % light hydrotreated distillate, 30 wt % light hydrotreated naphtha	blend of aliphatic and cyclic hydrocarbons	hydrotreated light naphtha distillate
FP [°C]	4^a^	<23^a^	>61^a^	>61
BP [°C]	100–155	>32	170–300	180–245
*D* [kg/m^3^] at 15 °C	730	706	771–871, 793^b^	700–900, 773^b^
LHV [MJ/kg]	44.925^14^	45.16^14^	N.A.	N.A.
			35.6^c^	35.2^c^
VP [kPa] 20 °C	2.04	47.57	<0.1;	N.A.;
			0.051^d^	0.07^d^
TOX: rat, oral, LD_50_ [g/kg]^e^	1–10 mg/L^f^	25	>5	2

a Closed cup flash point.

b Determined in this study, measured at 20 °C; LHV: lower heating value; N.A. not available. FP: flash point. BP: boiling point; D: density.

c Determined in this study; VP: vapor pressure.

d Calculated in this study (see the Supporting Information).

e TOX: toxicity measured as LD_50_: lethal dose.

f Ecotoxicity tested on fish, 96 h exposition (LC_50_).

This study aims to provide an environmental evaluation of selected commercially available LFs and possible biomass-based candidates, analyzing safety (FP and VP), energetic (LHV), toxicity (TOX), and environmental viewpoint (evaporation rate measurements and environmental sustainability based on ethanol equivalent (EE_4_)). To achieve this, the missing physicochemical properties of LFs and their biomass-based alternatives are determined (measured or calculated) and analyzed. Open cup flash points (OCFPs), densities, and evaporation rates are measured for Landmann, Terracotta, GVL, EL, and the mixtures of EtOH in the case of the latter two. Higher heating values (HHVs) of Landmann and Terracotta are measured, and LHVs are calculated based on the measurements. Finally, multicriteria analysis on four fossil-based conventional lighter fluids (Ronsonol, Zippo, Landmann, and Terracotta) and six selected biomass-based chemicals (γ-valerolactone, ethyl-levulinate, ethanol, *n*-butanol, γ-valerolactone 90% v/v and ethanol 10% v/v, and ethyl-levulinate 90% v/v and ethanol 10% v/v mixtures) is used to determine the best LF in terms of FP, BP, TOX, VP, and EE_4_ (Table 3).

**Table 3:** Physicochemical Properties of Selected Biomass-Based Compounds

lighter fluid	γ-valerolactone^24^	ethyl-levulinate	EtOH	*n*-BuOH
chemical formula	C_5_H_8_O_2_	C_7_H_12_O_3_	CH_3_CH_2_OH	C_4_H_9_OH
FP [°C]	81; 96^25^^a^	85^26^–94,^27^ 90^28^	13^29^	29–37,^30^ 35^31^
BP [°C]	207.5,^25,32^	205.8^33^	78^34^	117.7^31^
*D* [kg/m^3^] at 20 °C	1057; 1043^14^	1009.7^14^	789^35^	810^30,31^
LHV [MJ/kg]	26.46^36^	24.34^28^	26.9^37^^b^	36.02 ± 0.27^38^
VP [kPa] 20 °C	0.01^14^	0.02^14^	5.85^14^	0.667^39^
TOX: rat, oral, LD_50_ [g/kg]	8.8;^40^	>5^41^	7.060^42^	0.79^43^
	66.75^44^ > 5^45^			

a Open cup flash point.

b Using 1 Btu = 1.05506 kJ; 1 US gallon = 3.78541 and 1 L EtOH = 0.789 kg.

## 2 Results and Discussion

### 2.1 Evaluation from a Safety Point of View

**Figure 1:**
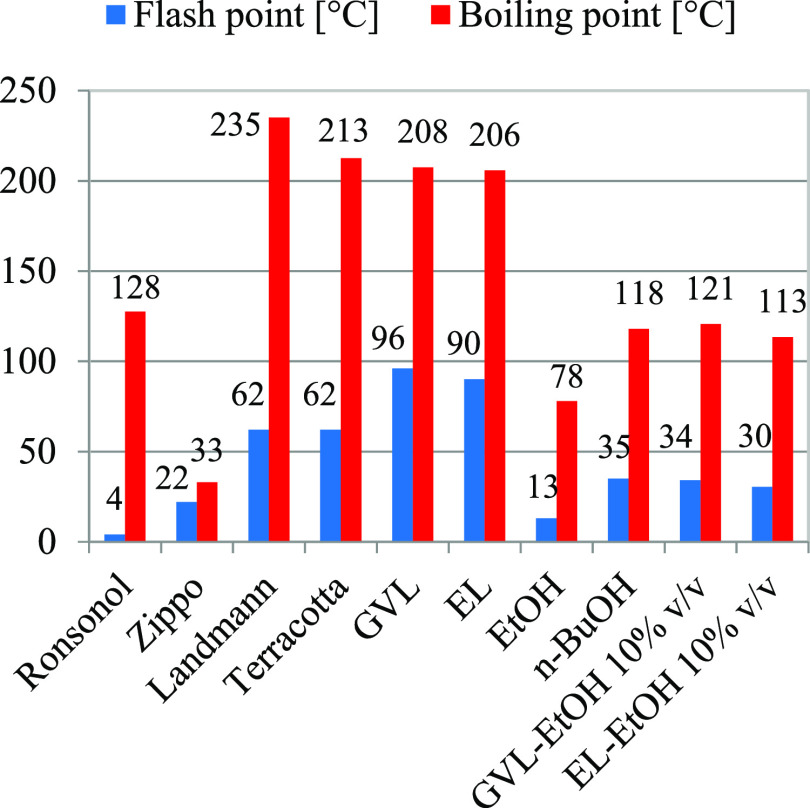
Flash points [°C] and boiling points [°C] of LFs.

As bioderived components are oxygenates, their mixtures are strongly nonideal and their properties, especially phase equilibrium-related properties, such as the boiling points of these mixtures, were calculated according to the UNIQUAC thermodynamic model, implementing the model parameters determined by Havasi et al.^46^ Considering FPs (Figure 1, blue bars), the lowest value belongs to Ronsonol and the highest to GVL, forecasting a very safe use of GVL. Still, the evaporation rate of mixture elements can likely be different and thus could lead to misleading values for flash points. In this case, it is better to measure the FPs of mixtures or estimate them by FP models. CCFP values were calculated for the GVL 90% v/v and EtOH 10% v/v mixture and the EL 90% v/v and EtOH 10% v/v mixture by using a model developed by Torabian and Sobati^47^ (see the detailed calculation in Supporting Information S2), and very low values of 34 and 30 °C were obtained, respectively.

OCFPs of GVL mixed with EtOH 10% v/v and EL mixed with EtOH 10% v/v were aimed to be measured with a Cleveland-type flash-point measuring device. Due to their low FP values, they were unmeasurable; therefore, lower ethanol-containing mixtures were determined instead. As shown in Table 4, a 5% v/v addition of EtOH can lower the pure GVL’s FP of 96 °C by ca. 20 °C, resulting in 75.33 °C as a mixture OCFP value; another 2% v/v increase of EtOH in the mixture further lowered it by 20 to 54.67 °C. Due to the very intensive evaporation of ethanol in the mixture, higher ethanol-containing mixtures could not be measured with this method.

**Table 4:** Measured Open Cup Flash Points of Mixtures as Biomass-Based Lighter Fluid Candidates

mixture	*T*_1_ [°C]	*T*_2_ [°C]	*T*_3_ [°C]	*T*_av_ [°C]	*p* [kPa]
GVL-EtOH 5% v/v	76	75	76	75.33	102.2
GVL-EtOH 7% v/v	55	55	53	54.67	102.2
EL-EtOH 5% v/v	56	55	56	55.67	101.0

Considering boiling points (Figure 1, red bars), the UNIQUAC thermodymanic model^46^ is used to calculate GVL—ethanol 10% v/v and EL—and ethanol 10% v/v mixtures. In the case of Zippo, the lowest value is provided as >32 °C; thus, 33 °C is selected as a representative value and shown in the graph, which seems to be the lowest one. When ranges are provided as boiling points in the case of Ronsonol, Landmann, and Terracotta, the average of the range is considered and shown in the graph. *n*-BuOH, GVL—ethanol 10% v/v and EL—and ethanol 10% v/v mixtures have a BP close to Ronsonol, but pure GVL, and EL are on the same scale as Landmann and Terracotta.

From an environmental point of view, evaporation acts as a key phenomenon in estimating volatiles’ emissions into the air during the preparation stage of the barbecue. Without US-type LFs (namely, Ronsonol and Zippo), evaporation tests are carried out for Landmann, Terracotta LFs, GVL, EL, and their mixtures with 10% v/v ethanol.

**Figure 2:**
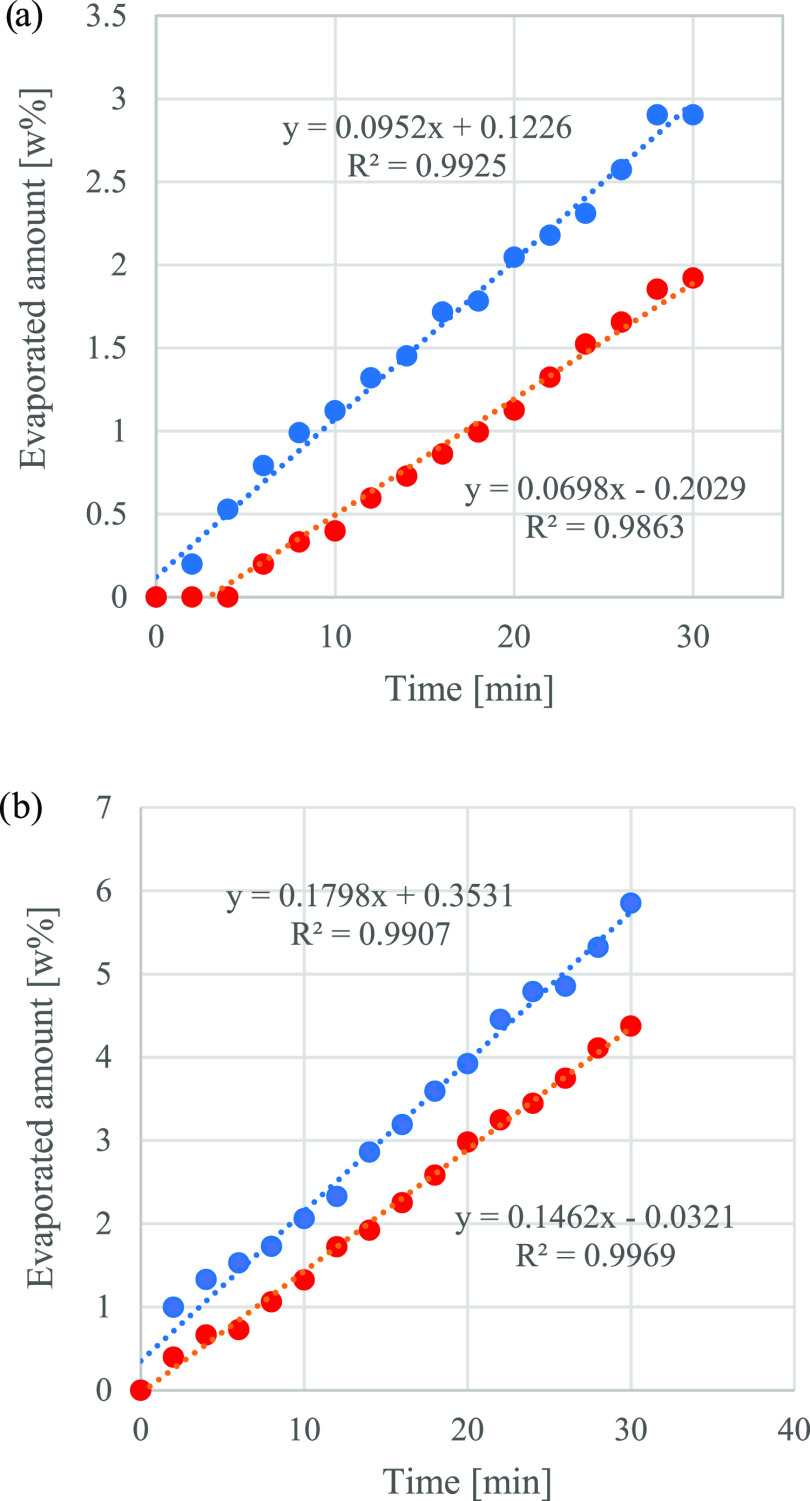
(a) Evaporated amount in wt % of Landmann (red solid circle) and Terracotta (blue solid circle) LFs at 30 °C and (b) evaporated amount of Landmann (red solid circle) and Terracotta (blue solid circle) lighter fluids [wt %] at 50 °C.

Evaporation tendencies show a linear relationship with time (indicated by the fitted lines) in all cases, similar to that obtained by the US Testing.^48^ The evaporation tendency of Terracotta is slightly higher than that of Landmann’s both at 30 and 50 °C (Figure 2a,b). The highest slope is obtained for Terracotta LF at 50 °C as 0.1798 wt %/min, representing only one-third of the evaporation rate determined by US Testing (see eq 1).^48^ The US Testing company estimated the emission of volatiles during the preparation of grilling (i.e., emission of evaporation origin) by sprinkling 103 mL of LF on 0.9 kg of charcoal briquettes at simulated outdoor conditions of an average day (50% relative humidity and 26.7 °C). Based on their measured data,^48^ a linear relationship is found between time and the evaporation of the liquid from charcoal
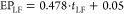
1where EP_LF_ [wt %] is the evaporated amount of LF, 0.478 [wt %/min],(48) and *t*_LF_ [min] is the charcoal soaking time of charcoal briquettes in LF.

Although no direct comparison can be made, the evaporation rate derived from our measured data at 30 °C is 1 order of magnitude lower for Landmann and Terracotta than for a US lighter fluid with unknown ingredients.

**Figure 3:**
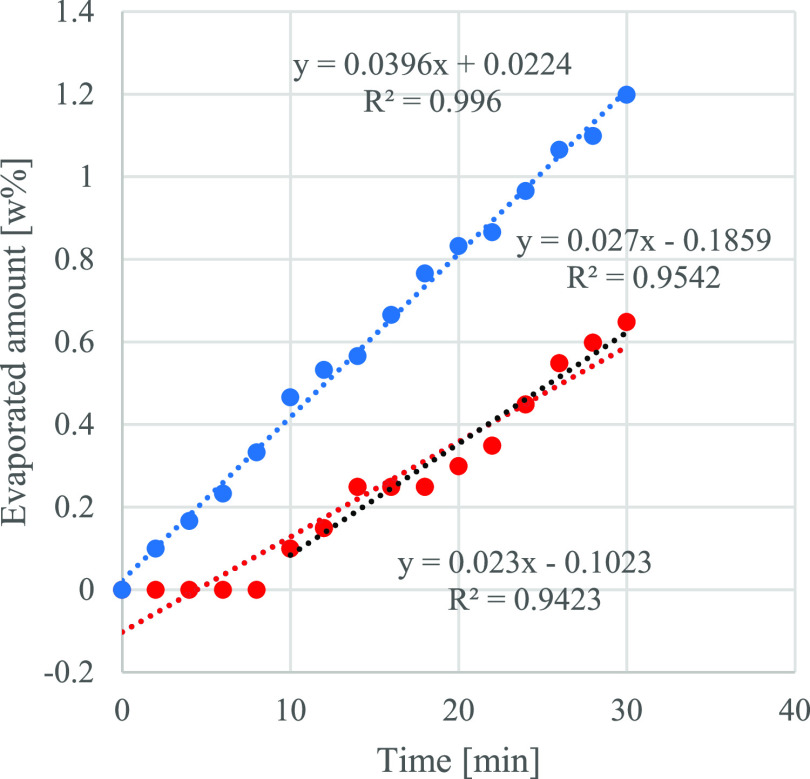
Evaporated amount of GVL (blue solid circle) and EL (red solid circle) (wt %) as a function of time at 50 °C.

**Figure 4:**
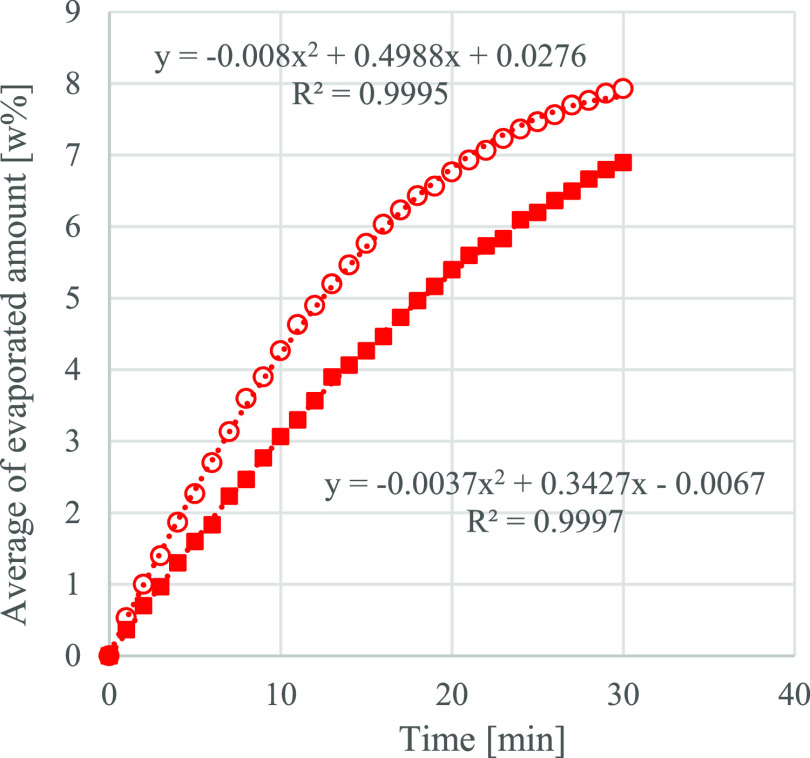
Average of the evaporated amount [wt %] of the EL 90% v/v and EtOH 10% v/v mixture at 30 °C (red solid square) and 50 °C (red circle) in the function of time.

**Figure 5:**
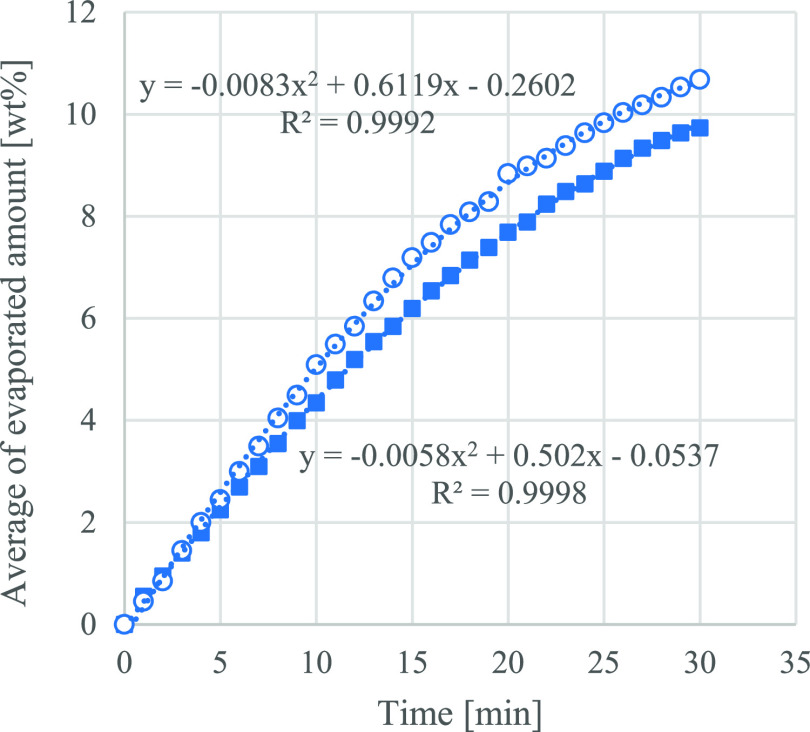
Average of the evaporated amount [wt %] of the GVL 90% v/v and EtOH 10% v/v mixture at 30 °C (blue solid square) and 50 °C (blue circle) in the function of time.

In the case of GVL and EL, practically no evaporation could be observed at 30 or 40 °C, and their values did not reach 0.35% after 30 min (see Supporting Information Figure S3). Thus, the measured data only at 50 °C are shown in Figure 3. It can be stated that EL requires a kind of lag phase in the first 10 min when no measurable change could be observed in mass. After this lag-phase, evaporation shows a linear relationship with measuring time, while GVL’s evaporation tendency is in good correspondence with those of hydrocarbon-based LFs’ but represents another order of magnitude lower evaporation rate (0.0396 wt %/min) than measured by US Testing for commercial LF (0.478 wt %/min). The significance of very low evaporation tendency prognoses a very safe use of biomass-based alternative LFs and a more difficult ignition simultaneously. It was previously proven that a small amount of ethanol addition enhances ignition, which was—on the other hand—an essential component in a future biomass-based LF.^14^ In further experiments, the evaporation rates of GVL 90% v/v—EtOH 10% v/v and EL 90% v/v—and EtOH 10% v/v are determined, and it is proven that the additional ethanol initiates the evaporation of the mixture; however, in the absence of gas analysis, the proportion of EL and GVL in the vapor is not known. According to the VPs of single components and evaporation rates of pure GVL and EL, it can be assumed that ethanol is the dominant compound evaporated. Adding ethanol to GVL and EL conspicuously changes the evaporation rate from linear to second order at 30 and 50 °C (Figures 3, 4 and 5).

**Figure 6:**
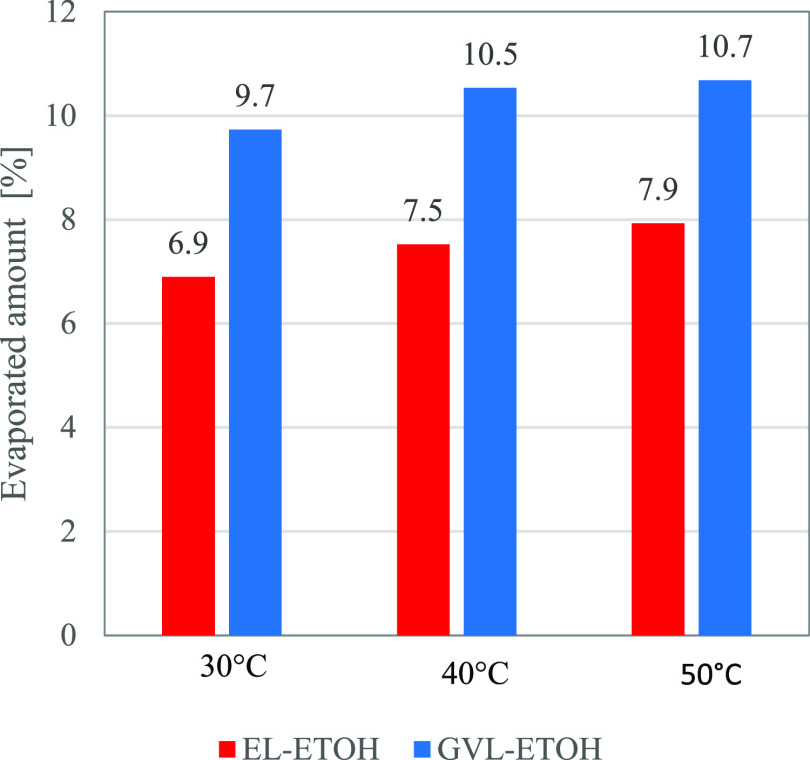
Evaporated amounts [%] of the mixtures of GVL and EL mixed with ethanol 10% v/v at different temperatures after 30 min.

On comparing the evaporated amounts of ethanol-containing mixtures (Figure 6), it is visible that almost 10 times more LFs are volatilized in the same time interval compared to pure GVL and EL.

It is worth noting that the addition of 10% v/v ethanol increases the evaporated amount to a similar level in the case of EL 90% v/v—ethanol 10% v/v or an even higher value in the case of GVL 90% v/v—and ethanol 10% v/v than those obtained for Landmann and Terracotta LFs.

Since the evaporation rate is closely related to VP, it is worth determining the VPs of Landmann and Terracotta LFs when knowing their exact compositions and their related Antoine constants. After identification of the components of Landmann and Terracotta LFs by GC-MS, VPs of the single components are calculated (see Supporting Information S1), and then the VP of a hydrocarbon mixture can be calculated according to Dalton’s equation
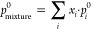
2where *x_i_* is the molar ratio of compound *i* and *p*_*i*_^0^ is the VP of compound *i* at 20 °C.

**Figure 7:**
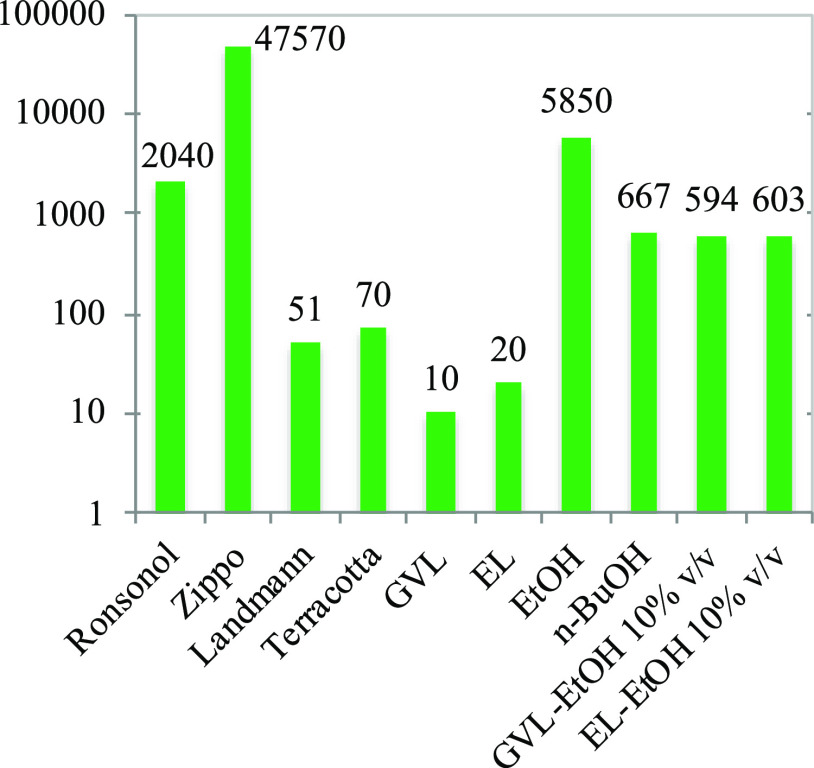
Vapor pressures [Pa] at 20 °C of lighter fluids and their biomass-based surrogates on a logarithmic scale.

VPs of LFs can vary in a wide range; therefore, logarithmic scaling is used for the representation and comparison of LFs (Figure 7). The VPs of pure GVL and EL are very low but in the same range as those of Landmann and Terracotta; all are below 100 Pa. Volatility of the biomass-based LF surrogates can be increased by 1 order of magnitude when mixing them with 10% v/v EtOH, making them comparable with *n*-BuOH, which is a constituent of a patented lighter fluid.^13^ Ronsonol-type LF and pure ethanol are the next in the ranking, exhibiting a 1000 Pa-scale VP, and the highest VP belongs to Zippo (47.57 kPa). Based on the VP analysis, Zippo, composed of 70 wt % light hydrotreated distillate, represents the highest VP and can lead to high volatility and emission, so it is better to select a biomass-based component mixed with ethanol.

### 2.2 Evaluation from an Energetic Point of View

LHV can provide information on the heat released during combustion related to a specific amount of fuel, namely, 1 kg of LF. When the flue gas is cooled to the initial temperature, vapor content is condensed, the heat of vapor condensation (*q*_cond_) is added to LHV, and HHV can be determined. LHV is lower when combusting a fuel containing carbon–oxygen or oxygen–hydrogen bonds, such as in the case of all biomass-based LFs (they are in the range of 24 and 36 MJ/kg) than in the case of hydrocarbon mixtures (see Tables 2 and 3). It is worth noting that no data were available for Landmann and Terracotta LFs; therefore HHVs of these LFs were measured with a Junkers-type calorimeter. Air used for combustion is saturated with water vapor, and after cooling the flue gas, the vapor is condensed; thus, the heat released during combustion is known as HHV. By knowing the amount of liquid water, LHV can be calculated. As shown in Table 5, LHVs of Landmann and Terracotta are close to *n*-butanol but ca. 10 MJ/kg lower than those of Ronsonol and Zippo and ca. 10 MJ/kg higher than those of GVL, EL, or pure EtOH.

**Table 5:** Higher and Lower Heating Values of Lighter Fluids

lighter fluid	HHV [MJ/kg]	*q*_cond_ [MJ/kg]	LHV [MJ/kg]
Landmann	36.4	0.7	35.6
Terracotta	35.8	0.6	35.2

### 2.3 Evaluation Based on Toxicity

**Figure 8:**
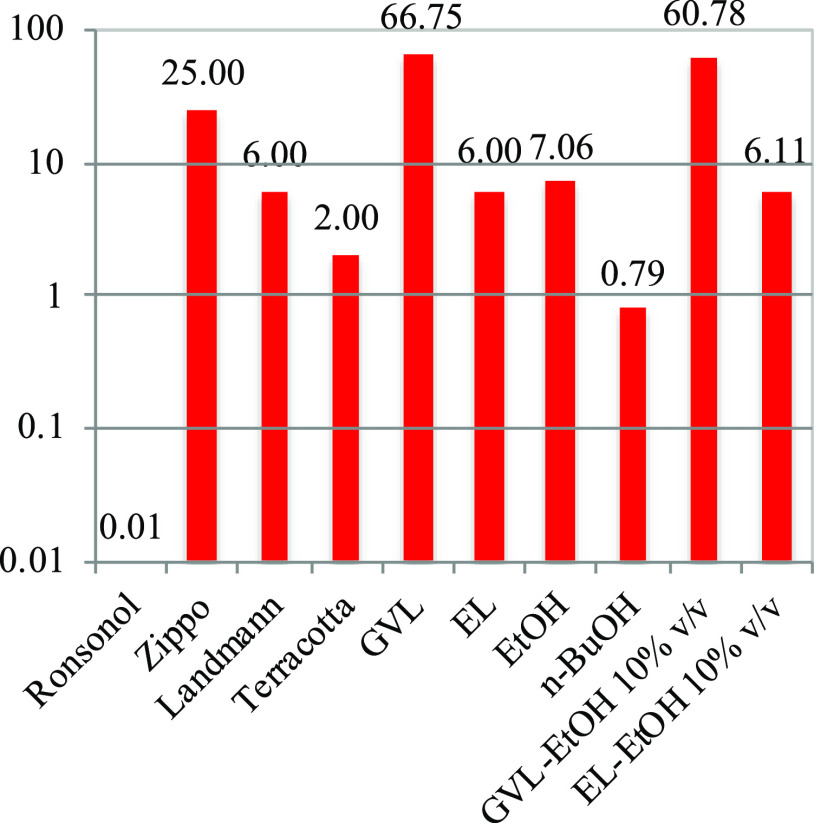
Oral toxicity (rat, LD_50_) [g/kg] of lighter fluids and their biomass-based surrogates.

Toxicological information is an essential part of safety data sheets because it indicates the health issues concerning dermal, oral, or inhalation contact. During the preparation phase of grilling, LF can spill on the skin, it can be swallowed by accident if the bottle is left uncovered, or it can evaporate and thus be inhaled. These are the main risks of LF usage. Oral toxicity values are available for all LFs and biomass-based surrogates, and LD_50_ values are collected and compared (Figure 8). LD_50_ is measured on rats and equals the amount of LF related to 1 kg of body mass if taken orally; half of the entities die. LD_50_ also considers the difference in the body weight. GVL is likely digestible since it is derived via carbohydrate conversion and represents an extremely high value (66.75 g/kg), while the most consumed biomass-based compound—EtOH—shows 1 order of magnitude lower value, i.e., 7.06 g/kg. Assuming a linear correlation between toxicity of single compounds (EL, GVL and EtOH), a weighted average accounts for LD_50_ values of mixtures such as EL 90% v/v—ethanol 10% v/v or GVL 90% v/v— ethanol 10% v/v. It is worth noting that no LD_50_ was available for Ronsonol. Further, 0.01 mg/kg is an estimation based on the ecotoxicity tested on fish: 96 h exposition as lethal concentration (LC), i.e., LC_50_ = 1–10 mg/L. Based on toxicity, it can be concluded that GVL or its mixture with ethanol is a consumable chemical and can be used as biomass-based nontoxic LFs.

### 2.4 Evaluation Based on the Ethanol Equivalent

Horváth and coworkers introduced the ethanol equivalent (EE) as a sustainability metric that can act as a conversional tool between biomass-based and fossil-based feedstocks because bioethanol is a representative of a fuel and a carbon-based chemical simultaneously.^2^ Considering the energy requirement of bioethanol production with an ethanol return on ethanol (ERoE^49^) value, EE_4_ as a real ethanol equivalent can be calculated, which indicates that the ethanol equivalent includes the use of 1 unit of bioethanol to produce four units of bioethanol. Thus, the abbreviation EE_4_ used in this paper shows a four output/input bioethanol ratio or efficiency. Using lower heating values, EE_4_ values of fossil-based and biobased LFs were calculated in our previous work, assuming an annual consumption of 5000 t LF.^14^ Calculations now are extended to *n*-BuOH as a recently proposed LF candidate and Ronsonol, Zippo, Landmann, and Terracotta commercial LFs. As shown in Table 6, the lowest EE_4_ value can be obtained for GVL, followed by GVL 90% v/v and EtOH 10% v/v, while the *n*-BuOH’s value is relatively close to the hydrocarbon-based LFs’. Because EE_4_ is calculated based on energy content, the highest EE_4_ is expected for the LF with the highest LHV, i.e., Zippo.

**Table 6:** Ethanol Equivalents of Different LFs

lighter fluids	LHV [kJ/kg]	energy-based EE [kT]	energy-based EE_4_ [kT]	refs
Ronsonol	44,925	7.535	9.419	
Zippo	45,160	7.574	9.468	
Landmann	35,600	5.971	7.464	
Terracotta	35,200	5.904	7.38	
GVL	26,465	4.439	5.549	(14)
EtOH	29,673	4.977	6.221	(14)
GVL 90% v/v and EtOH 10% v/v	26,712	4.48	5.6	(14)
EL	30,988	5.197	6.496	(14)
EL 90% v/v and EtOH 10% v/v	30,884	5.18	6.475	(14)
*n*-BuOH	36,022	6.041	7.551	

### 2.5 Multicriteria Analysis Based on Environmental Aspects

**Figure 9:**
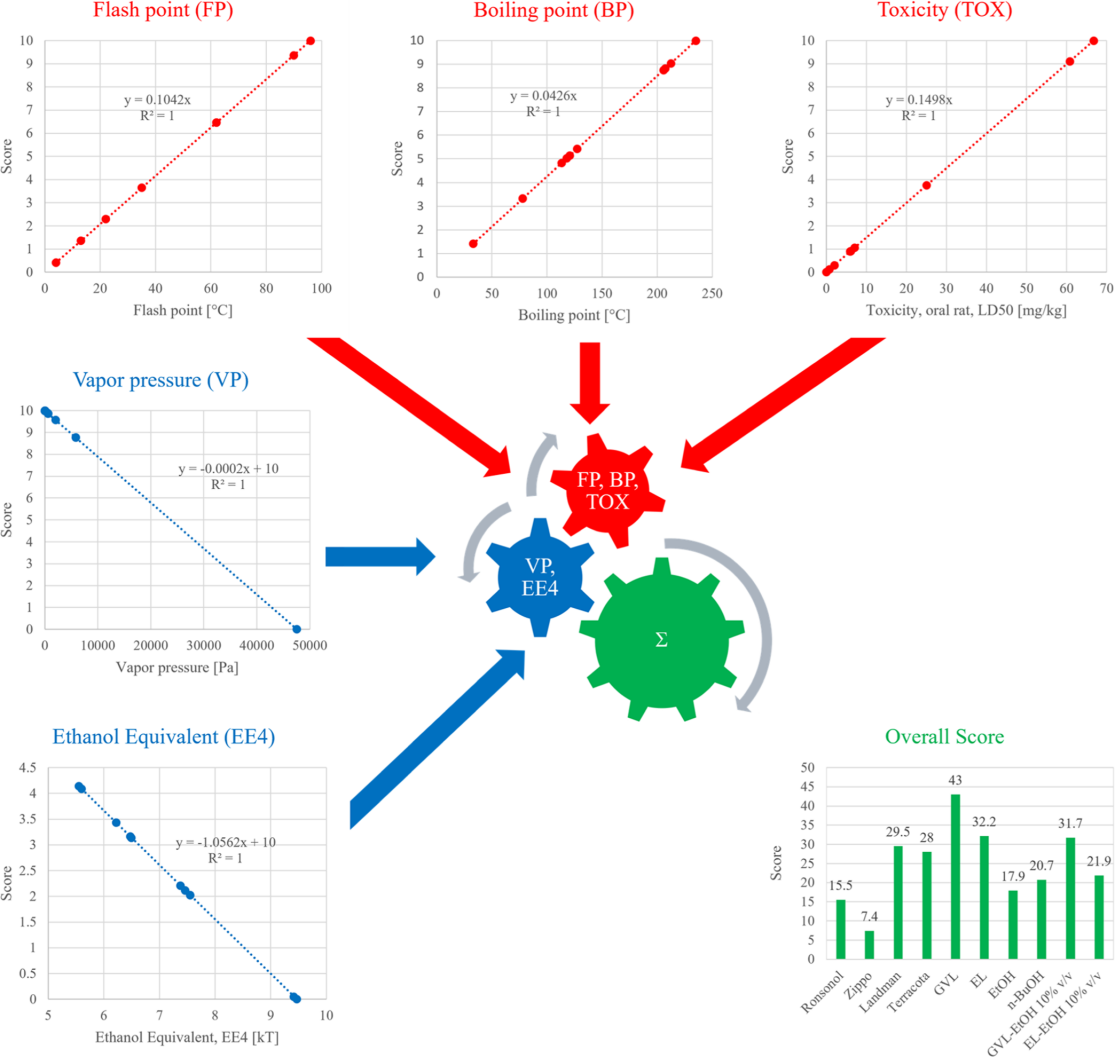
Multicriteria analysis of lighter fluids.

Multicriteria analysis is carried out to obtain an environmental evaluation of four fossil-based conventional lighter fluids (Ronsonol, Zippo, Ladmann, and Terracotta) and six biomass-based chemicals (GVL, EL, EtOH, *n*-BuOH, GVL 90% v/v and EtOH 10% v/v, and EL 90% v/v and EtOH 10% v/v mixtures) based on FP, BP, TOX, VP, and EE_4_. At first, parameters are grouped into two: FP, BP, and TOX are parameters that represent the “the higher is better” principle; when the FP is high, the LF is not ignitable very easily and is less volatile, resulting in a reduced evaporated amount, thus reducing the risk of inhalation. A linear correlation is set according to this principle, and a score between 0 and 10 is applied for the range representing all LFs, i.e., a 0 score is set for the lowest value, and 10 is accounted for the highest value obtained. The second group consisted of VP and EE_4_ possessing a reversed evaluation; the “lower is the better” principle is used in this case. As the VP is low, low volatility, low evaporation rate, and reduced risk of inhalation are expected. In accordance with the previous scoring, a linear correlation with a negative slope is adapted to these data, thus creating a weighted score for each parameter: the lowest value is scored as 10 and the highest value is scored as 0. The principle of this multicriteria analysis, the equations adapted to each category, and the overall scores are shown in Figure 9 and Table 7 and can help in the selection of the best LF based on the mentioned parameters. Analyzing the LFs by each parameter, the highest score is dedicated to GVL when considering FP. Landmann possesses the highest BP, while GVL is an orally consumable chemical to rats in a comparison of toxicity values. The highest scores go to GVL when considering both VP and EE_4_, respectively, so it can be concluded that GVL wins in the case of four of the five studied parameters. The highest overall score is obtained for GVL, which is in correspondence with our previous findings.^14^

**Table 7:** Overall Scores Obtained from Multicriteria Analysis are Given in a Descending Order

lighter fluids	FP	BP	TOX	VP	EE_4_	sum of scores
GVL	10.0	8.8	10.0	10.0	4.1	43.0
EL	9.4	8.8	0.9	10.0	3.1	32.2
GVL 90% v/v and EtOH 10% v/v	3.5	5.1	9.1	9.9	4.1	31.7
Landmann	6.5	10.0	0.9	10.0	2.1	29.5
Terracotta	6.5	9.0	0.3	10.0	2.2	28.0
EL 90% v/v and EtOH 10% v/v	3.1	4.8	0.9	9.9	3.2	21.9
*n*-BuOH	3.6	5.0	0.1	9.9	2.0	20.7
EtOH	1.4	3.3	1.1	8.8	3.4	17.9
Ronsonol	0.4	5.4	0.0	9.6	0.1	15.5
Zippo	2.3	1.4	3.7	0.0	0.0	7.4

## 3 Conclusions

Despite the strained efforts of researchers, engineers, and politicians, our society still greatly depends on fossil resources: 82.2% of the world’s primary energy consumption was based on oil, natural gas, and coal in 2021.^50^ While we are not able to completely avoid the usage of fossil resources in the energy sector now, more focus should be given to the replacement of fossil resources as raw materials. The chemical industry started turning to biomass-based consumer products 20 years ago,^51^ and some of the suggestions have been realized and become industrially viable. Still, it has also been pointed out that the volume is a crucial factor in replacement and biomass conversion. Lighter fluids are consumer products used only at the kiloton scale, which need to possess similar physicochemical properties as fuels used in the energy sector. This study has revealed that low-volume-used lighter fluids can be replaced by biomass-based alternatives in terms of safety, toxicological, and environmental viewpoints. A multicriteria analysis using physicochemical properties (flash point, boiling point, toxicity, vapor pressure, and ethanol equivalent) pointed out that GVL as a renewable chemical could be a good alternative (out of four fossil-based conventional lighter fluids and six selected biomass-based surrogates). It can be estimated from the evaporation tests that emission of the evaporation origin is low when using GVL or GVL 90% v/v and EtOH 10% v/v mixtures and even lower for EL and EL 90% v/v and EtOH 10% v/v mixtures compared to commercial LFs. The significance of a very low evaporation tendency prognoses a safe use of biomass-based alternative LFs. In practical usage, however, ignition, combustion experiments, flue gas, and emission analyses are also required.

## 4 Experimental Section

### 4.1 Chemicals

γ-Valerolactone (γ-valerolactone or GVL, CAS No. 108-29-2), levulinic acid (CAS No. 12 3-76-2), pure ethanol (EtOH, CAS No. 64-17-5), and benzyl-alcohol (used for calibration, CAS No.: 100-51-6) were obtained from Sigma-Aldrich Ltd., Budapest, Hungary. Landmann and Terracotta lighter fluids were purchased from the OBI Hardware Store.

### 4.2 Apparatus and Procedure

**Vapor pressures** of Landmann and Terracotta at 20 °C and given temperatures were calculated according to the Antoine equation by knowing the exact composition of these LFs. Component analysis was executed by GC-MS, and the Antoine constants of identified compounds or the surrogates were used from the ChemCAD or the NIST database. Corresponding equations and parameters are provided in Supporting Information S1 andTables S1 and S2, and eqs S1–S3.

**Closed cup flash point** values were calculated for the GVL 90% v/v and EtOH 10% v/v mixture and the EL 90% v/v and EtOH 10% v/v mixture by using a model developed by Torabian and Sobati and presented in Supporting Information S2 and eqs S4–S6. **Flash point** determination was executed on a Cleveland-type flash-point measuring device, which is suitable for determining the open-air flash point, the so-called open cup flash point (OCFP). Benzyl-alcohol, having an FP of 101 °C^52^ at atmospheric pressure, was used for the calibration. The accuracy of the OCFP measurement was calculated by using the calibration fluid: 102.3 °C was determined as FP at atmospheric pressure (*p* = 101.2 kPa), representing a relative error of 1.28%. It is worth noting that 104.4^53^ and 105 °C^54^ were also reported as OCFP of benzyl-alcohol. Detailed measuring procedures and equipment are shown in Supporting Information S2 and Figure S1.

**The evaporation rate** was determined in a drying furnace at 30, 40, and 50 °C. LFs were poured into a premeasured Petri dish, covering its whole surface. 10.00 ± 0.1 mL was used for GVL, EL, and their mixtures with EtOH, and 5.00 ± 0.1 mL for commercial LFs. After adjusting the temperature and reaching a constant value, LFs in premeasured Petri dishes were placed into a furnace. The mass of the LF-containing Petri dish was measured every 2 min. Each experiment was repeated three times, and averages were considered during the evaluation. The evaporation rate was calculated on the measured mass of LFs versus time. The detailed measurement procedure and equipment are shown in Supporting Information S3 and Figure S2. Evaporated amounts of GVL and EL [wt %] at 30 and 40 °C are shown in Figure S3.

**Densities** of Landmann and Terracotta LFs, GVL, and EL were measured in a 50 mL pycnometer at 20 °C with an accuracy of ±0.0015 mL.

**Higher heating values** of hydrocarbon mixtures such as Landmann and Terracotta LFs were measured in a Junkers-type calorimeter. LFs were placed under a 0.3 barg overpressure in a fuel tank, evaporated through a nozzle, and mixed with saturated air to combust. **A lower heating value** could be derived by knowing the temperature increase and the mass rate of cooling water and subtracting vapor condensation’s enthalpy. The detailed measurement procedure and equipment are shown in Supporting Information S4 and Figure S4 and eqs S7–S10.

## Supporting Information Available

Vapor pressures (S1); composition of the Terracotta lighter fluid and vapor pressures of components at 20 °C (S1.1); components of the Terracotta lighter fluid identified by GC-MS, relating Antoine constants and the calculated vapor pressures (*p*^0^) of each compound (Table S1); composition of the Landmann lighter fluid and vapor pressures of components at 20 °C (S1.2); components of the Landmann lighter fluid identified by GC-MS, relating Antoine constants and the calculated vapor pressures (*p*^0^) of each compound (Table S2); model, apparatus, and procedure of flash point determination (S2); molar fractions of compounds in the GVL 90% v/v and EtOH 10% v/v mixture and the EL 90% v/v and EtOH 10% v/v mixture (Table S3); Cleveland-type flash-point measuring device (Figure S1); apparatus and procedure of evaporation rate determination (S3); weighing scale, stopwatch, and thermostat used for evaporation tests at 30, 40, and 50 °C (Figure S2); evaporated amount of GVL and EL [wt %] as a function of time at 30 and 40 °C (Figure S3); apparatus and procedure of higher heating value determination (S4); and Junkers-type calorimeter (Figure S4) (PDF)

## Dedication

^†^ This work is dedicated to the memory of Professor István T. Horváth, who passed away tragically in 2022 and would have been 70 years old on August 6, 2023. Following his idea, this research work began and deals with the use of GVL as a lighter fluid.

## Abbreviations

BP: boiling point
D: density
ECHA: European Chemicals Agency
EE: ethanol equivalent
EL: ethyl-levulinate
ERoE: ethanol return on ethanol
EtOH: ethanol
FP: flash point
GVL: γ-valerolactone
HHV: higher heating value
IPCs: initial platform chemicals
LC: lethal concentration
LD_50_: lethal dose
LF: lighter fluid
LHV: lower heating value
MSDS: material safety data sheet
*n*-BuOH: *n*-butanol
OCFP: open cup flash point
TOX: toxicity
VP: vapor pressure
