# Supplementary material for: Environmental Sustainability of Lighter Fluids†
Source: ACS Omega. 2024 Jan 17;9(4):4277–86. doi: 10.1021/acsomega.3c05242 (PMC10832035; doi:10.1021/acsomega.3c05242)
Supplement: Supplementary file 1 — ao3c05242_si_001.pdf [file ao3c05242_si_001.pdf]

Supplementary Information to

Environmental sustainability of lighter fluids

Edit Cséfalvay and Viktória Kovács*

Department of Energy Engineering, Faculty of Mechanical Engineering, Budapest University of Technology and Economics, Műegyetem rkp. 3., H–1111 Budapest, Hungary

S1 Vapor pressures S2

S1.1 Composition of Terracotta lighter fluid and vapor pressures of components at 20°C S2

S1.2 Composition of Landmann lighter fluid and vapor pressures of components at 20°C S3

S2 Model, Apparatus and Procedure of Flash point Determination S3

S3 Apparatus and Procedure of Evaporation Rate Determination S6

S4 Apparatus and Procedure of Higher Heating Values Determination S7

*Corresponding author: Department of Energy Engineering, Faculty of Mechanical Engineering, Budapest University of Technology and Economics, Műegyetem rkp. 3., Budapest, Hungary, H-1111. Tel: +36 1 463 2559, e-mail: csefalvay.edit@gpk.bme.hu

S1 Vapor pressures

S1.1 Composition of Terracotta lighter fluid and vapor pressures of components at 20°C

Table S1 Components of Terracotta lighter fluid identified by GC-MS, relating Antoine constants and the calculated vapor pressures (p^0) of each compound

Component	A	B	C	D	E	T_{min} [K]	T_{max} [K]	p^0 [Pa]	p^0 [bar]	Eq. Nr	Ref.
Decane	0.21021	440.616	-156.896			243.49	310.59		3.630E-03	S1	[1]
4-methyl-decane [a]	4.21961	1640.288	-72.933			273	462.37		4.615E-03	S1	[2]
2,5-dimethyl-nonane	54.276	-3368	-5.261	0.0000086	2	85.44	369.82	1711682		S2	[3]
2-methyl-decane	4.21961	1640.288	-72.933			273	462.37		4.615E-03	S1	[4]
5-izobutyl-nonane [b]	118.27	-11432	-13.769	5.9641E-06	2	267.76	675.8	52		S2	3
Undecane	195.83	-12914	-27.327	0.024106	1	247.57	638.76	317		S2	3
2-methyl-undecane	84.867	-9634.7	-8.6541	5.096E-18	6	215.15	655	157		S2	3
dodecane	128.8	-11593	-15.385	7.0698E-06	2	263.57	658.2	118		S2	3

[a] replaced by 2-methyl-decane, [b] replaced by tridecane

Vapor pressure of a given component at given temperature was calculated according to different types of Antoine equations (S1 and S2):

$$\log_{10}(p^0) = A - \frac{B}{C+T} \tag{S1}$$

where:

- p^0 — vapor pressure of the given molecule [bar]
- A, B, C — Antoine-constants
- T — temperature at which vapor pressure is calculated [K], now T=293.15 K.

A ChemCAD software uses Antoine-constants for the natural logarithmic function at a given temperature:

$$\ln(p^0) = A + \frac{B}{T} + C \cdot \ln(T) + D \cdot T^{\mathrm{E}}. \tag{S2}$$

where:

- p^0 — vapor pressure of the given molecule, [Pa]
- A, B, C — Antoine-constants
- D, E — Further Antoine-constants
- T — Temperature at which vapor pressure is calculated T=293.15 K.

S1.2 Composition of Landmann lighter fluid and vapor pressures of components at 20°C

Table S2 Components of Landmann lighter fluid identified by GC-MS, relating Antoine constants and the calculated vapor pressures (p^0) of each compound

Component	A	B	C	D	E	T_{min} [K]	T_{max} [K]	p^0 [Pa]	p^0 [bar]	Eq. Nr	Ref.
Decane	0.21021	440.616	-156.896			243.49	310.59		3.630E-03	S1	1
4-methyl-decane [a]	4.21961	1640.288	-72.933			273	462.37		4.615E-03	S1	2
2-methyl-decane	4.21961	1640.288	-72.933			273	462.37		4.615E-03	S1	2
5-izobutyl-nonane [b]	118.27	-11432	-13.769	5.9641E-06	2	267.76	675.8	52		S2	3
1-ethyl-1-methyl cyclohexane [c]	82.454	-7845	-8.8018	3.7436E-06	2	178.25	693.15	2232		S2	3
Undecane	195.83	-12914	-27.327	0.024106	1	247.57	638.76	317		S2	3
Hexadecane	82.454	-7845	-8.8018	3.7436E-06	2	178.25	693.15	2		S2	3
2-methyl-undecane	84.867	-9634.7	-8.6541	5.096E-18	6	215.15	655	157		S2	3
dodecane	4.10549	1625.928	-92.839			399.53	490.49		1.111E-03	S1	5

[a] replaced by 2-methyl-decane, [b] replaced by tridecane, [c] replaced by n-propyl-cyclohexane

Vapor pressures of Terracotta and Landmann lighter fluids (represented as $p^0_{mixture}$) were calculated according to Dalton equation, considering the weighted sum of each compound's vapor pressure:

$$p^0_{mixture} = \sum_i x_i \cdot p^0_i \quad \text{(S3)}$$

Where:

$p^0_{mixture}$ vapor pressure of lighter fluid [Pa]

x_i molar fraction of compound *i* in the lighter fluid [mol/mol]

p^0_i vapor pressure of compound *i* [Pa]

S2 Model, Apparatus and Procedure of Flash point Determination

Closed cup flash points values were calculated for GVL 90% v/v and EtOH 10% v/v mixture and EL 90% v/v and EtOH 10% v/v mixture by using a model developed by Torabian and Sobati. [6] Blending index for component *i*, namely GVL, EtOH and EL was calculated according to Eq. S4,

$$BI_{FP,i} = FP_i^{-1/X} \quad \text{(S4)}$$

where X= 0.0382, as determined for alcohol-acetate binary mixtures and FP_i is the closed cup flash point of component *i* in °C, namely 96°C and 90°C for GVL and EL, respectively. Having the

$BI_{FP,\,i}$ indices for the pure compounds and weighing them according to the molar fraction of each component, $BI_{FP,\,\text{GVL 90\% v/v and EtOH 10\% v/v}}$ and $BI_{FP,\,\text{EL 90\% v/v and EtOH 10\% v/v}}$ could be calculated according to Eq.s S5 and S6:

$$BI_{FP,\,\text{GVL 90\% v/v and EtOH 10\% v/v}} = \sum_{i\,=\,GVL}^{n\,=\,EtOH} x_i \cdot BI_{FP,i} \tag{S5}$$

$$BI_{FP,\,\text{GVL 90\% v/v and EtOH 10\% v/v}} = \sum_{i\,=\,EL}^{n\,=\,EtOH} x_i \cdot BI_{FP,i} \tag{S6}$$

Table S 3 Molar fractions of compounds in GVL 90% v/v and EtOH 10% v/v mixture and EL 90% v/v and EtOH 10% v/v mixture

Molar fraction	GVL 90% v/v	EtOH 10% v/v	EL 90% v/v	EtOH 10% v/v
x_i	0.846	0.154	0.787	0.213

By substituting the related molar fractions (Table S 3) and blending indices, closed cup flash points of binary mixtures could be calculated as 34 and 30°C for GVL 90% v/v and EtOH 10% v/v mixture and EL 90% v/v and EtOH 10% v/v mixture, respectively.

Flash point (FP) determination was executed on a Cleveland type flash point measuring device, which is suitable for determining the open-air flash point – so called open cup flash point (OCFP) – and ignition point of all flammable liquids and petroleum derivatives having a flash point higher than 50°C. The device consists of a sample container, an ignition head and ignition arm, a thermometer, a thermometer holder, and a heater, which is electrically heated. Benzyl-alcohol having a FP of 101°C [7] at atmospheric pressure was used for the calibration. Accuracy of OCFP measurement was calculated by using the calibration fluid: 102.3°C was determined as FP at atmospheric pressure (p = 101.2 kPa) representing a relative error of 1.28%. Noteworthy that 104.4°C [8] and 105°C [9] were also reported as OCFP of benzyl-alcohol.

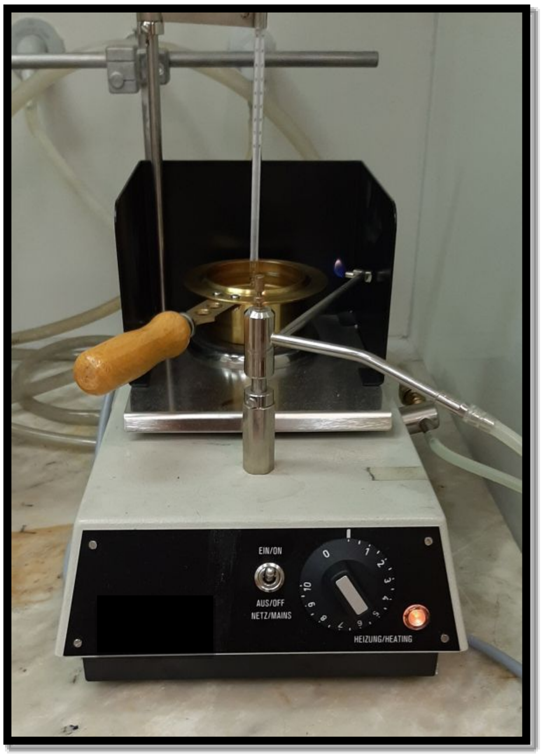

Figure S-1 Cleveland type flash point measuring device

Procedure

- proper cleaning and drying of the sample container;
- filling the sample holder up to the mark (70 mL of liquid);
- placing the thermometer in the correct position;
- lighting a support flame and setting the flame size;
- the initial heating rate is set at 15 °C/min; then reduced to 5.5°C/min when approaching the expected flash point;
- moving the support flame over the liquid must be started 30°C below the expected flash point;
- support flame must be moved towards the sample for every 2°C increase in temperature until the flash point is reached;

- Flash point is the temperature at which ignition is detected when the ignition lever is inserted.

S3 Apparatus and Procedure of Evaporation Rate Determination

Evaporation rate was determined in a drying furnace at temperatures of 30°C, 40°C and 50°C. Lowest temperature was set at 30°C agreeing that most commonly outdoor BBQs are carried out around this air temperature. On a sunny day charcoal can collect sunshine and be heated up, therefore 50°C was selected as the highest temperature for evaporation of LFs. LFs were poured into a Petri dish covering its whole surface. 10.00 ± 0.1 mL was used for GVL, EL and their mixtures with ethanol, and 5.00 ± 0.1 mL for commercial LFs. After adjusting the temperature of the furnace, LFs in a premeasured Petri dish were placed into the furnace. Mass of the LF-containing Petri dish was measured in every two minutes. Each experiment was repeated three-times, and averages were taken into account during evaluation. Evaporation rate was calculated on the measured mass of LFs versus time.

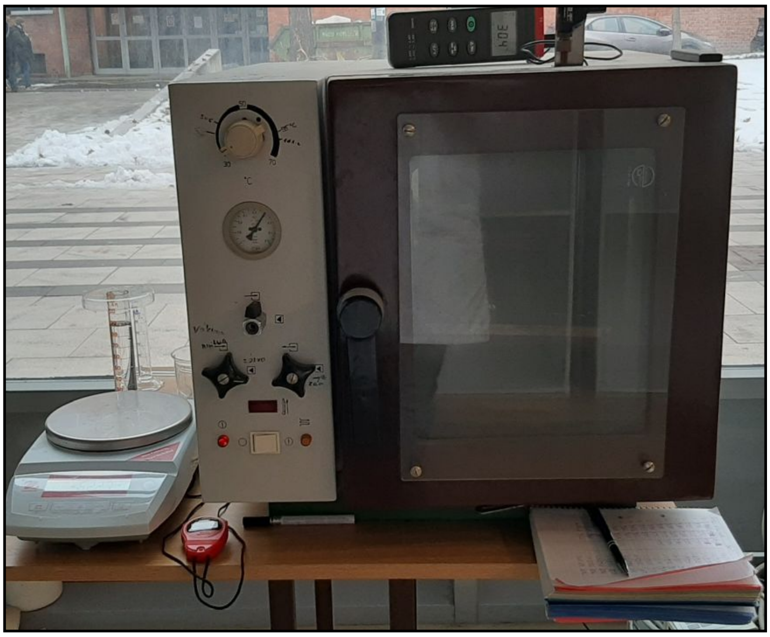

Figure S-2 Weighing scale, stopwatch and thermostat used for evaporation tests at 30, 40 and 50°C

Evaporations of pure GVL and EL at 30 nor 40°C began after 10 minutes of conditioning. These values were not significant and remained below 0.35% (Figure S-3).

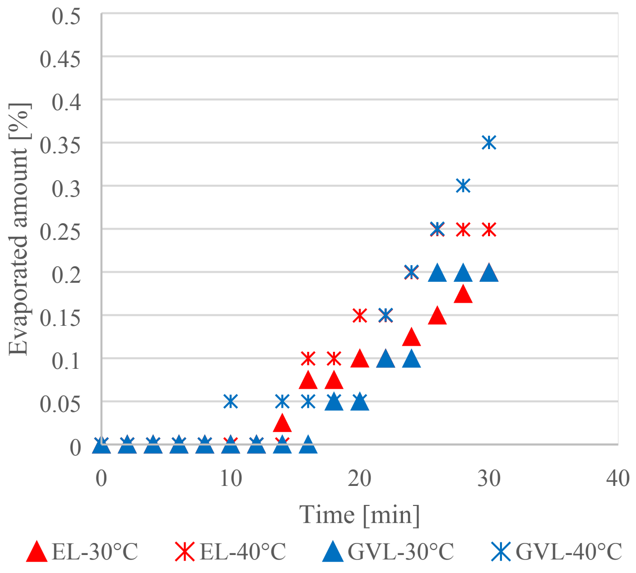

Figure S-3 Evaporated amount of GVL and EL [wt%] as a function of time at 30°C and 40°C

S4 Apparatus and Procedure of Higher Heating Values Determination

Higher Heating Values of hydrocarbon mixtures such as Landmann and Terracotta LFs were measured in a Junkers-type calorimeter (Figure S-4). LFs were placed under 0.3 barg overpressure in a fuel tank, then evaporated through a nozzle and mixed with air to combust. To reduce measurement errors, air was saturated with water vapor. Combustion was carried out in a heat exchanger cooled with cooling water thus the enthalpy of combustion was absorbed by cooling water, moreover water vapor originating from the saturated air and combustion of hydrogen containing lighter fluid were condensed. By knowing the temperature increase and mass rate of cooling water higher heating value could be calculated easily, then by subtracting the enthalpy of vapor condensation **lower heating value** could be derived.

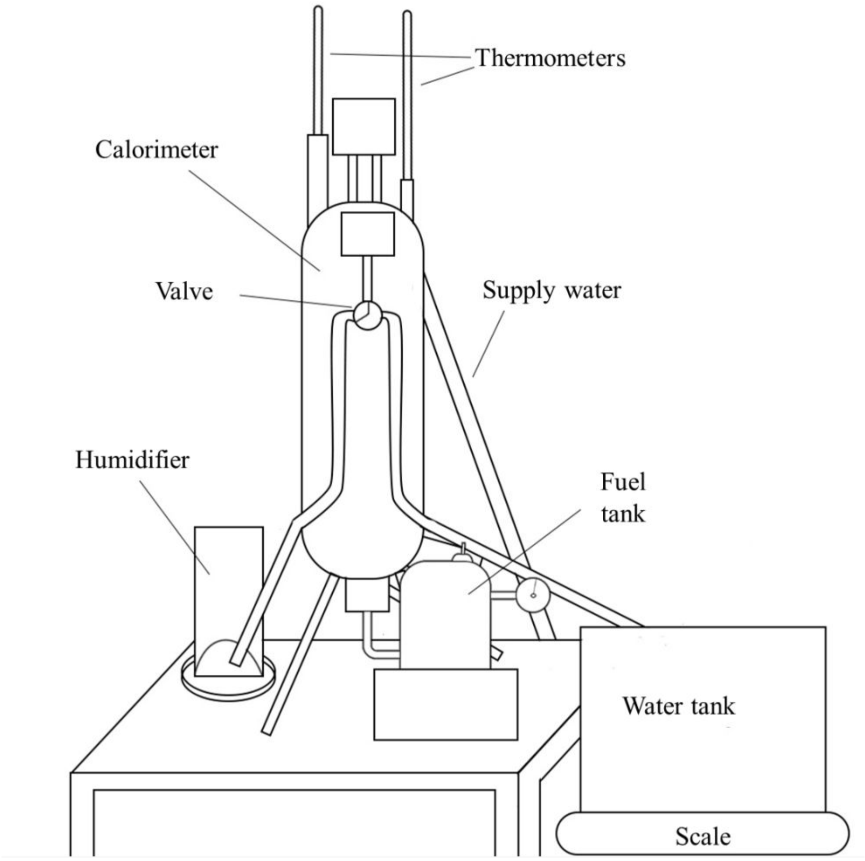

Figure S-4 Junkers type calorimeter

When the heat balance equation of the calorimeter is rearranged, higher heating value can be calculated according to Eq. S7.

$$HHV \cdot \Delta m_{\mathrm{LF}} = c_{\mathrm{w}} \cdot m_{\mathrm{w}} \cdot (t_{\mathrm{w,\,out}} - t_{\mathrm{w,\,in}}) \tag{S7}$$

$$HHV = \frac{c_{\mathrm{w}} \cdot m_{\mathrm{w}} \cdot (t_{\mathrm{w,\,out}} - t_{\mathrm{w,\,in}})}{\Delta m_{\mathrm{LF}}} \tag{S8}$$

Where

HHV	Higher Heating Value [kJ/kg]
Δm_{LF}	mass of the consumed lighter fluid during the experiment [kg]
c_w	Isobaric specific heat capacity of water, c_w=4.1868 kJ/(kg·K)
m_w	Mass of cooling water [kg]

$t_{w,\,out}$ Average outlet temperature of cooling water in steady state [°C]

$t_{w,\,in}$ Average inlet temperature of cooling water in steady state [°C].

$$q_{\text{cond}} = m_{\text{cond}} \cdot r \tag{S9}$$

Where:

q_{cond} Enthalpy of condensation [kJ]

m_{cond} Mass of condensed vapor (from saturated air [kg]

r Enthalpy of water evaporation, r=2500 kJ/kg.

By subtracting the enthalpy of vapor condensation from the higher heating value **lower heating value** could be derived according to Eq. S10.

$$LHV = HHV - q_{\text{cond}}. \tag{S10}$$

where

LHV Lower Heating Value [kJ/kg].

References

(1) NIST database, webbook,

http://webbook.nist.gov/cgi/cbook.cgi?ID=C124185&Units=SI&Mask=4#Thermo-Phase

(2) NIST database, webbook,

http://webbook.nist.gov/cgi/cbook.cgi?ID=C6975980&Units=SI&Mask=4#Thermo-Phase

(3) *CHEMCAD*; 6.5.6.7502 Copyright 2008-2014; ChemStations Inc. 2017.

(4) NIST database, webbook,

http://webbook.nist.gov/cgi/cbook.cgi?ID=C6975980&Units=SI&Mask=4#Thermo-Phase

(5) NIST database, webbook,

http://webbook.nist.gov/cgi/cbook.cgi?ID=C112403&Units=SI&Mask=4#Thermo-Phase

(6) Torabian E., Sobati, M. A., New models for predicting the flash point of mixtures containing different alcohols, Process Saf. Environ. Prot., **2017**, *111*, 439-448.

(7) Material Safety Data sheet of Benzyl alcohol, https://www.merckmillipore.com/HU/hu/product/Benzyl-alcohol,MDA_CHEM-822259 (accessed on 30 January 2023)

(8) Lewis, R.J. Sr.; *Hawley's Condensed Chemical Dictionary* 15th Edition. John Wiley & Sons, Inc. New York, NY **2007,** 140. Cited in PubChem database https://pubchem.ncbi.nlm.nih.gov/compound/244#section=Flash-Point (accessed on 30 January 2023)

(9) O'Neil, M.J. (ed.). The Merck Index - *An Encyclopedia of Chemicals, Drugs, and Biologicals*. Cambridge, UK: Royal Society of Chemistry, **2013**, 198. Cited in PubChem database https://pubchem.ncbi.nlm.nih.gov/compound/244#section=Flash-Point (accessed on 30 January 2023)
